# Supplementary material for: Elucidating the Mechanism of Coumarin Homodimerization Using 3-Acetylcoumarin Derivatives
Source: Molecules. 2025 Feb 1;30(3):651. doi: 10.3390/molecules30030651 (PMC11820059; doi:10.3390/molecules30030651)

Supplementary materials

Elucidating the Mechanism of Coumarin Homodimerization Using 3-Acetylcoumarin Derivatives

Kristina B. Simeonova [1], Ana I. Koleva [1], Nevena I. Petkova-Yankova [1], Anna-Mariya R. Zlatanova [1], Vesela Lozanova [2], Rositca D. Nikolova [1,*] and Petko St. Petkov [1,*]

[1] Faculty of Chemistry and Pharmacy, Sofia University "St. Kliment Ohridski" 1164 Sofia, Bulgaria; ohks@chem.uni-sofia.bg (K.B.S.); ohak@chem.uni-sofia.bg (A.I.K.); nipetkova@chem.uni-sofia.bg (N.I.P.-Y.); an.zlatanova@gmail.com (A.-M.R.Z.)

[2] Department of Medicinal Chemistry and Biochemistry, Medicinal University of Sofia, 1431 Sofia, Bulgaria; vlozanova@medfac.mu-sofia.bg

* Correspondence: rnikolova@chem.uni-sofia.bg (R.D.N.); ppetkov@chem.uni-sofia.bg (P.S.P.); Tel.: +359-2-81-61-392 (R.D.N.); +359-2-81-61-433 (P.S.P.)

<u>List of content:</u>

1. General reaction scheme for the proposed radical and ionic mechanisms S2
2. Calculation for ionic mechanism S2-S3
3. NBO charges of the atoms S3
4. Initial and final geometries of the model system S4
5. NMR-data S5-S13

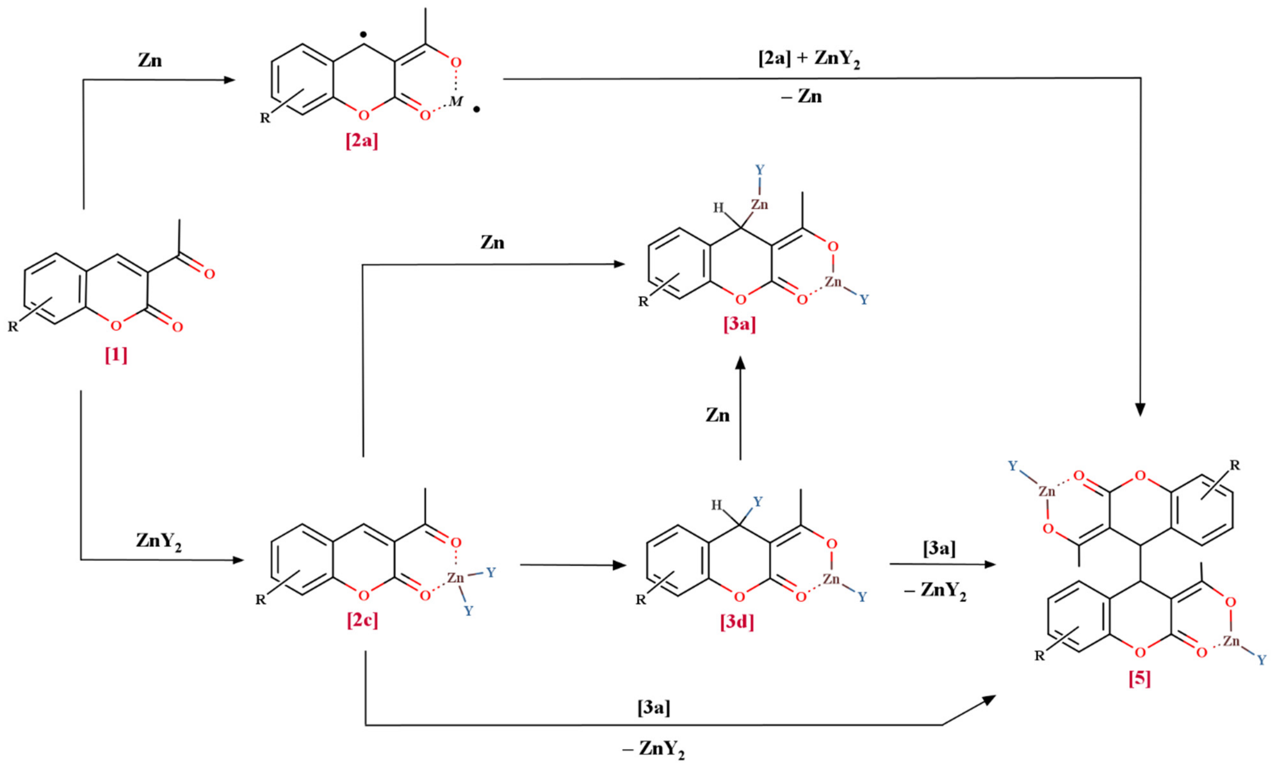

Figure S1: General reaction scheme for the proposed radical and ionic mechanisms for the formation of the homodimeric product.

In **Table S1** and **Table S2** are presented the calculated free energies for the formation of the proposed intermediates, as well as the homodimeric product, using the ionic reaction path.

Table S1: Calculated free energies for the formation of the intermediates and the homodimerization product with $ZnCl_2$ as a metal salt in PCM (THF) via the ionic reaction path. All energies are in [kJ/mol].

Structure	[1] → [2c]	[2c] → [3a]	[2c] → [3d]	[3d] → [3a]	[2c] → [5]	[3d] → [5]
**3-accoum**	20	–54	81	–136	–20	–101
**6-Cl**	–6	–62	77	–140	–20	–97
**6-OMe**	–15	–52	83	–135	–20	–103
**6-NO_2**	1	–76	68	–144	–22	–90
**7-OMe**	–19	–38	N/A	N/A	–8	N/A
**7-NO_2**	5	–89	66	–144	–21	–87
**7-Et_2N**	–21	–7	N/A	N/A	19	N/A
**8-OMe**	–4	–58	74	–132	–93	–93

Table S2: Calculated free energies for the formation of the intermediates and the homodimerization product with chloroacetic anhydride as a metal salt in PCM (THF) via the ionic reaction path. All energies are in [kJ/mol].

Structure	[1] → [2c]	[2c] → [3a]	[2c] → [3d]	[3d] → [3a]	[2c] → [5]	[3d] → [5]
**6-Cl**	42	–69	72	–141	–19	–91
**6-OMe**	28	–58	85	–143	–15	–99
**6-NO_2**	47	–87	62	–150	–22	–84
**7-OMe**	24	–38	N/A	N/A	–5	N/A
**7-NO_2**	47	–87	62	–149	–14	–77
**7-Et_2N**	27	–21	N/A	N/A	N/A	N/A
**8-OMe**	43	–53	89	–142	–70	–159

Table S3: NBO charges of the atoms in the lactone ring in the neutral forms of the 3-acetylcoumarin and its derivatives. 1 – O; 2-4 – C.

Structure	1	2	3	4
**3-accoum**	–0.510	0.794	–0.277	–0.058
**6-Cl**	–0.503	0.796	–0.245	–0.080
**6-OMe**	–0.505	0.792	–0.260	–0.064
**6-NO_2**	–0.498	0.798	–0.239	–0.079
**7-OMe**	–0.510	0.794	–0.285	–0.57
**7-NO_2**	–0.499	0.796	–0.219	–0.092
**7-Et_2N**	–0.517	0.793	–0.307	–0.063
**8-OMe**	–0.499	0.793	–0.259	–0.063

Table S4: NBO charges of the atoms in the lactone ring in the radical forms of the 3-acetylcoumarin and its derivatives. 1 – O; 2-4 – C.

Structure	1	2	3	4	Zn
**3-accoum**	–0.520	0.827	–0.345	–0.200	0.878
**6-Cl**	–0.515	0.827	–0.344	–0.195	0.881
**6-OMe**	–0.515	0.826	–0.344	–0.199	0.877
**6-NO_2**	–0.514	0.825	–0.344	–0.191	0.887
**7-OMe**	–0.520	0.826	–0.348	–0.209	0.878
**7-NO_2**	–0.503	0.831	–0.334	–0.125	0.900
**7-Et_2N**	–0.522	0.826	–0.344	–0.230	0.872
**8-OMe**	–0.508	0.828	–0.345	–0.203	0.877

Figure S2. Initial and final geometries of the investigated system for the formation of the biscoumarin via radical mechanism. **(a)** and **(b)** show the initial geometries; **(c)** and **(d)** show the geometries after optimization. As balls are presented C-4 atoms, as well as oxygen in the radicals, zinc and the molecule of the metal salt. Gray – Carbon; red – Oxygen; blue-gray – Zinc; green – Chlorine.

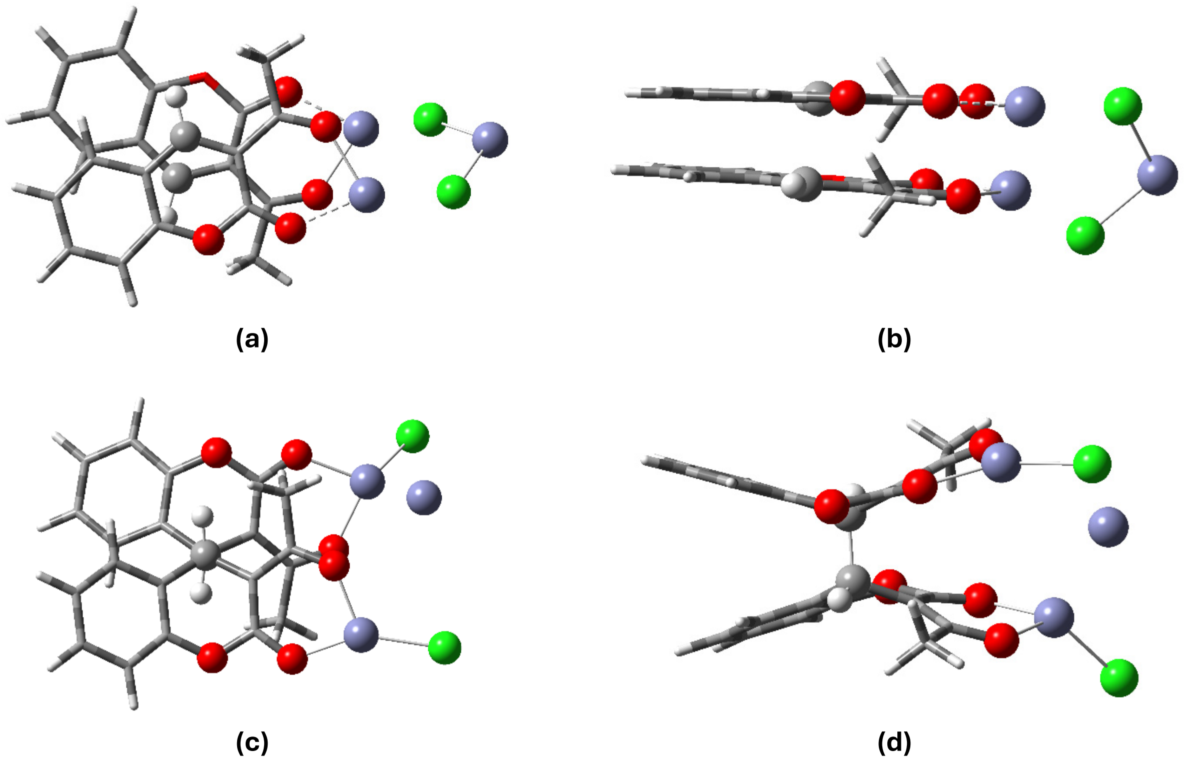

Table S5. NMR data from CPMAS NMR for compounds **2b-f** at 15 kHz.

Atom, ν [ppm]	2b	2d	2e	2f	2g
<u>C</u>OCH₃	179.2<br>177.2	178.7<br>177.4	181.8<br>181.5	179.6	180.0
COO	171.6	171.2	178.1	179.4	170.8
C-8a,8a’	146.5	147.6	150.7	150.5<br>150.1	149.5
C-6,6’	157.6<br>156.9	111.7	169. 5	169. 8	118.8
4xCH					130.6
C-7,7’	125.9<br>118.2	125.2	133.0<br>131.8	129.5<br>127.1	127.9
C-5,5’	117.9<br>117.5	125.2	126.8<br>126.2	126.8	125.3
C-4a	119.4	123.1	125.5<br>124.8		122.5
C-8,8’	111.2<br>109.3	140.9	120.4<br>119.1	119.4	117.8
C-3	95.3<br>92.4	95.7<br>92.5	93.8<br>91.0	95.0<br>91.1	95.3
O<u>C</u>H₃	55.6	56.2			
C-4	45.1<br>43.1	46.3<br>44.1	45.5<br>44.8	45.8<br>44.3	40.1
CO<u>C</u>H₃	17.8	18.1<br>16.7	19.2<br>17.9	19.3<br>17.4	17.7

Figure S3. CPMAS NMR spectra of compounds **2b-h**.

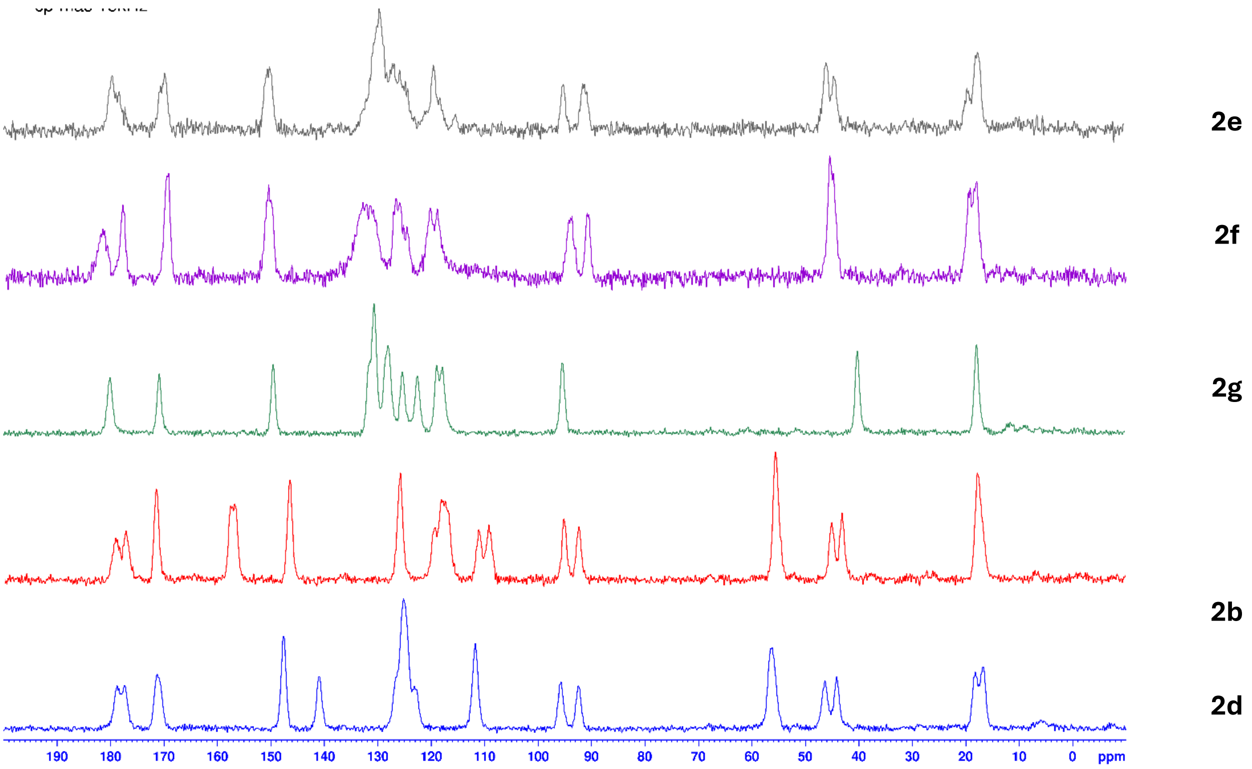

[1]**H NMR** spectrum of mixture of **2b**$_{\mathbf{A}}$ and **2b**$_{\mathbf{B}}$ in TFA-d.

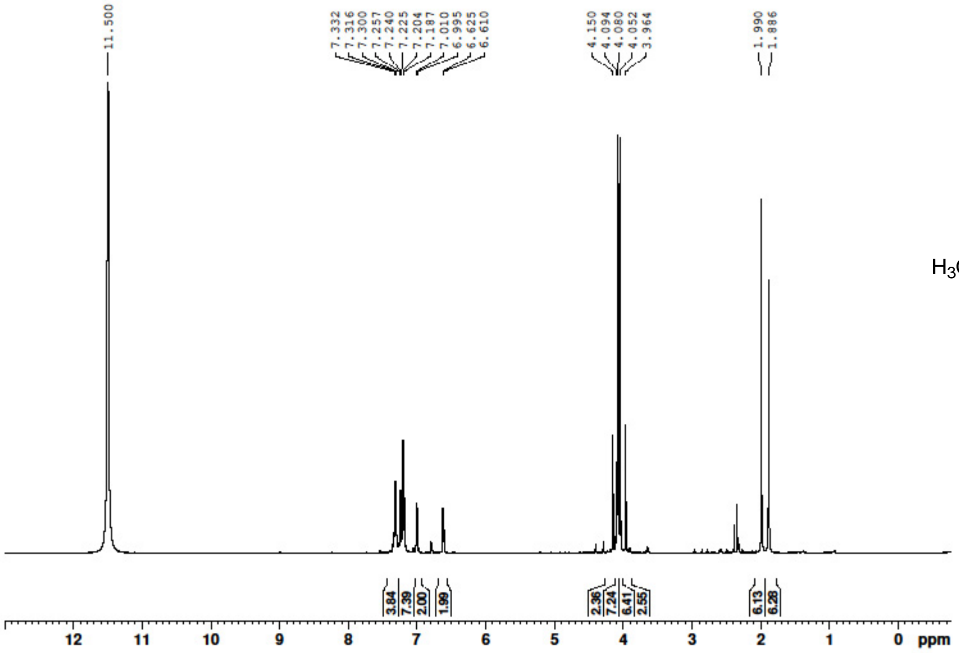

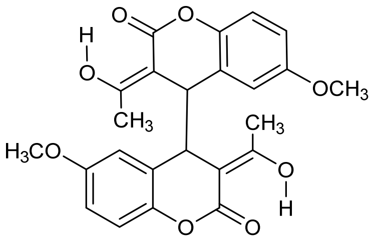

[13]**C NMR** spectrum of mixture of **2b**$_{\mathbf{A}}$ and **2b**$_{\mathbf{B}}$ in TFA-d.

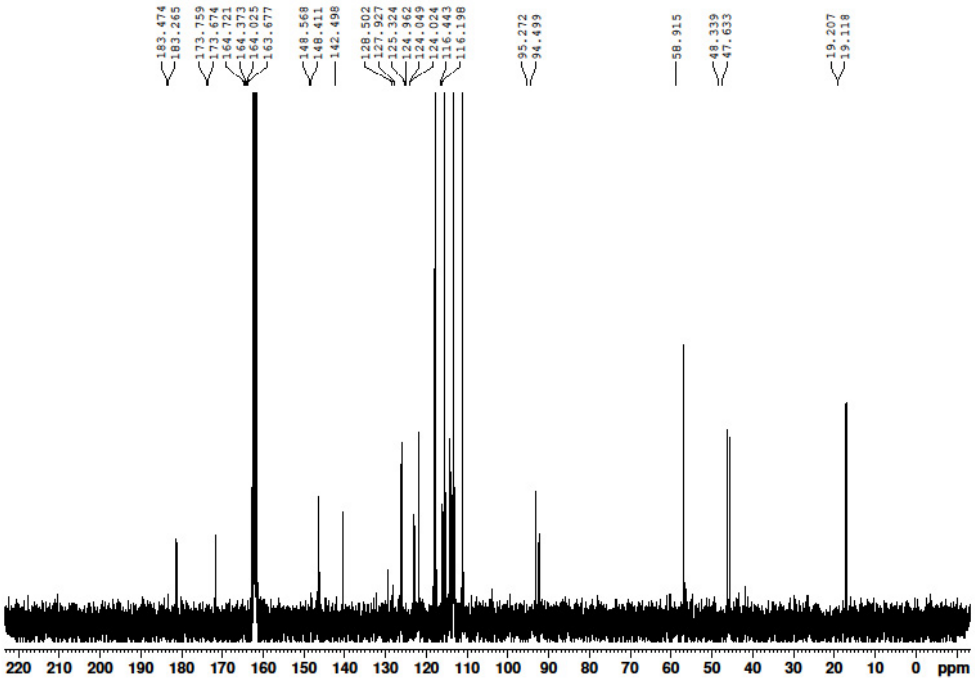

{^{1}H,^{13}C} **HSQC** spectrum of **2b**$_A$ and **2b**$_B$ in TFA-d.

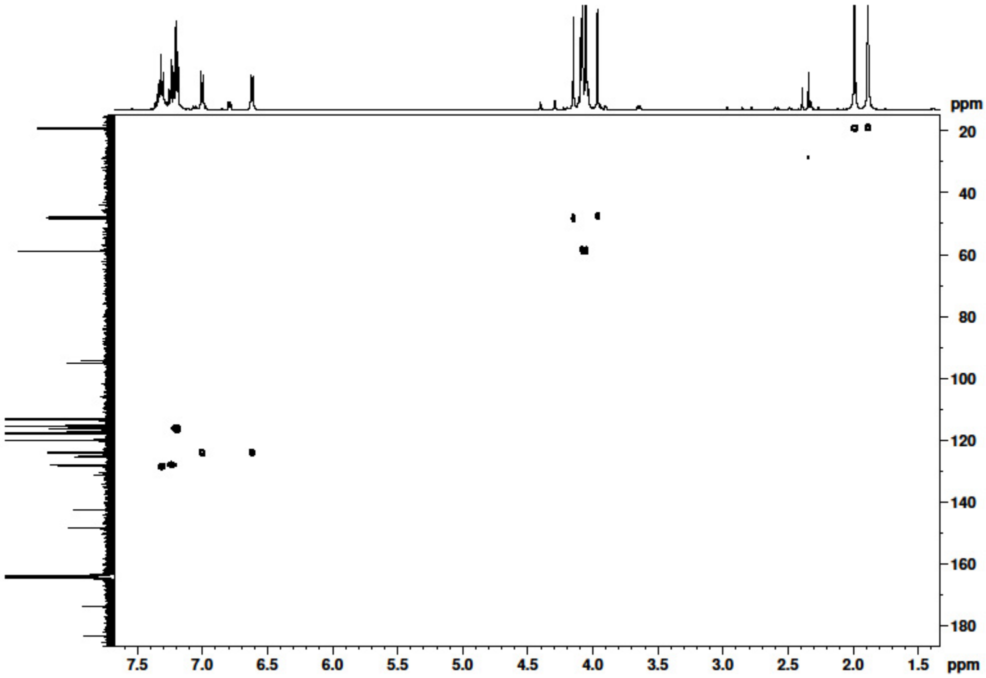

NOESY spectrum of **2b**$_A$ and **2b**$_B$ in TFA-d.

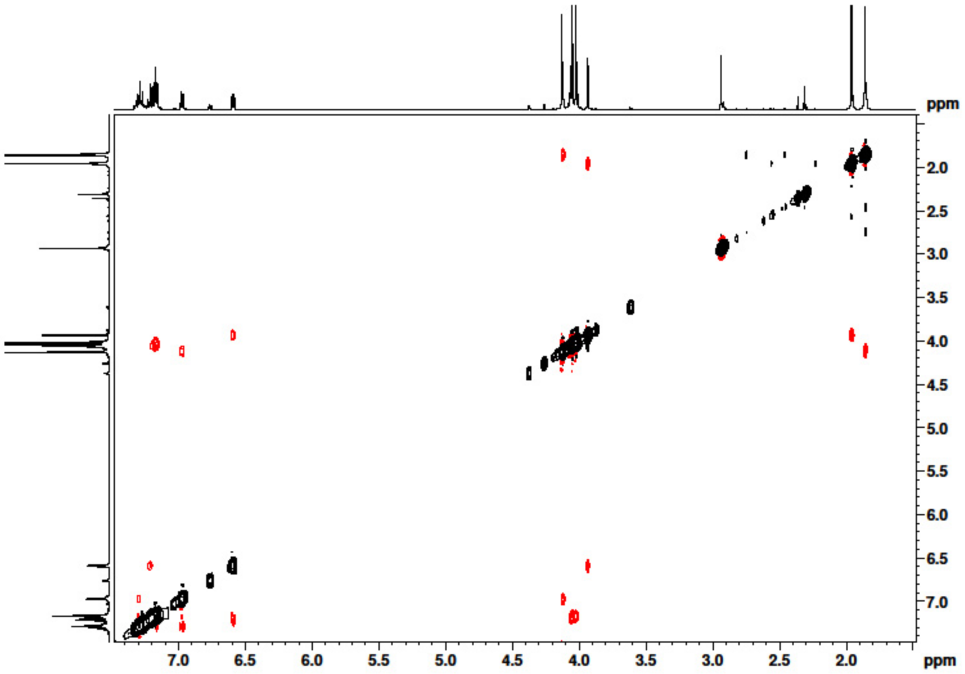

^{1}**H NMR** spectrum of mixture of **2d$_A$** and **2d$_B$** in TFA-d.

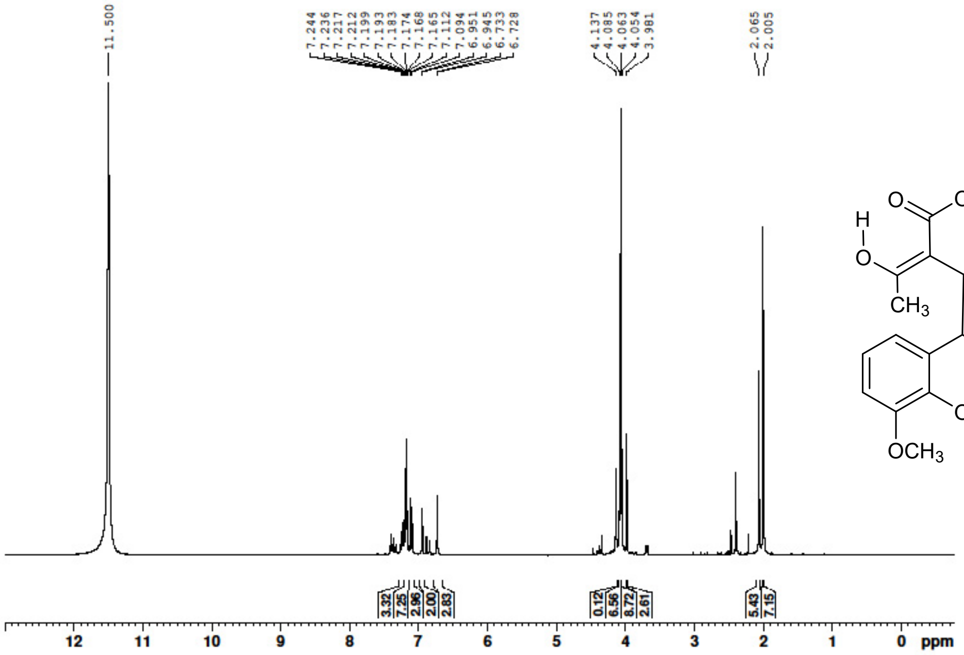

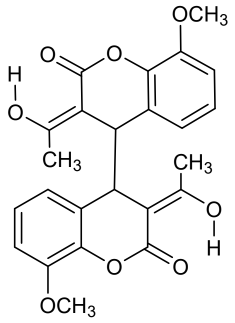

^{13}**C NMR** spectrum of mixture of **2d$_A$** and **2d$_B$** in TFA-d.

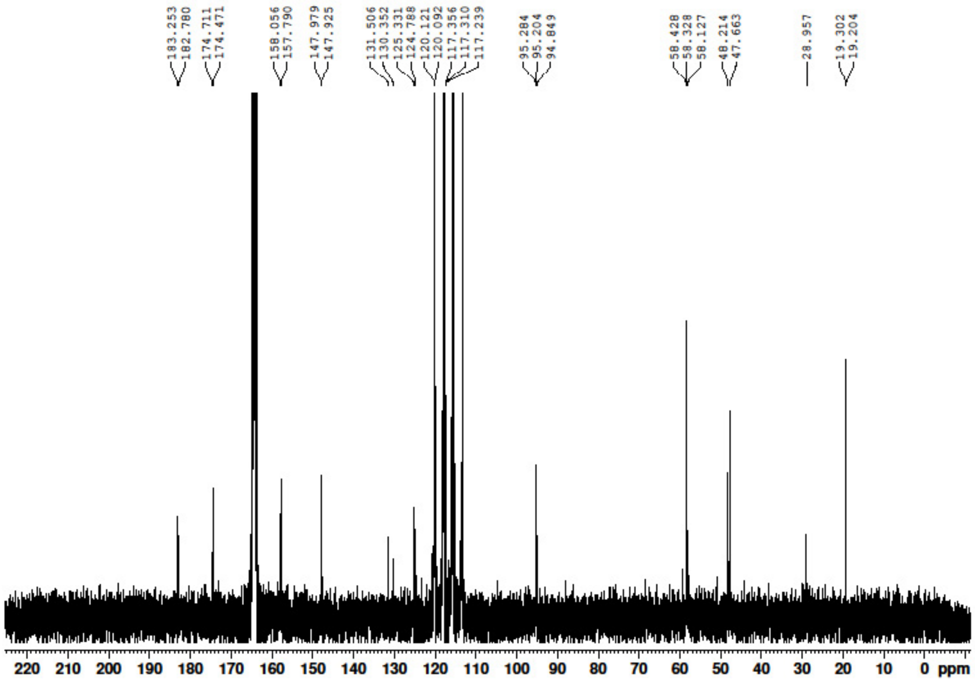

[1]**H NMR** spectrum of mixture of **2e**$_A$ and **2e**$_B$ in TFA-d.

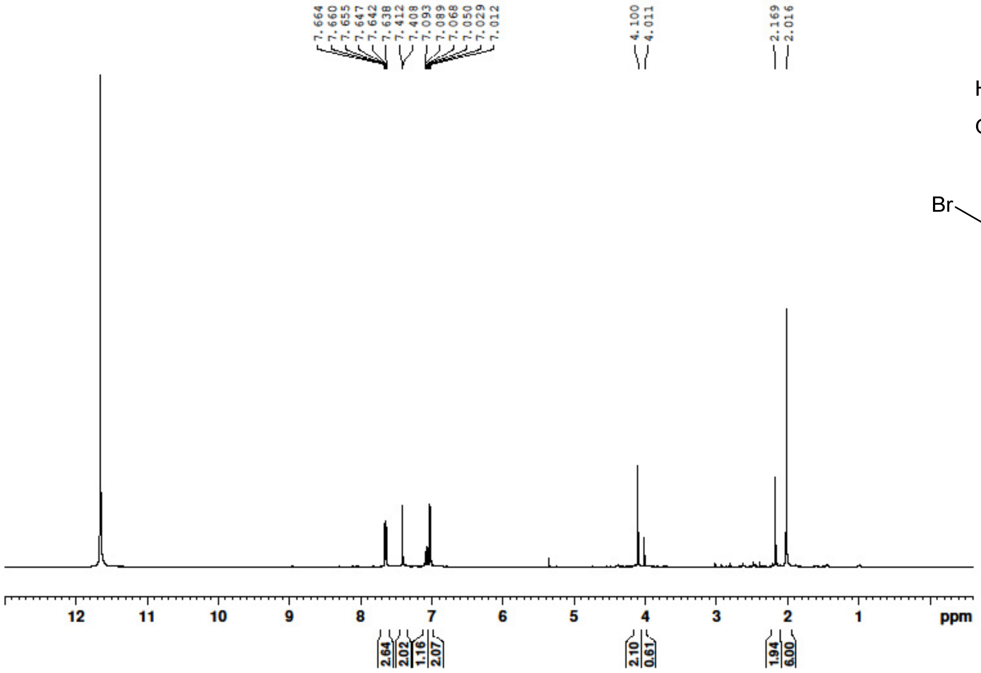

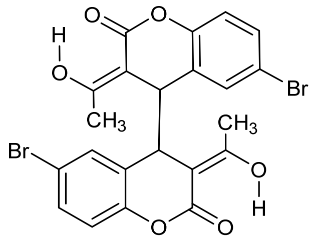

[13]**C NMR** spectrum of mixture of **2e**$_A$ and **2e**$_B$ in TFA-d.

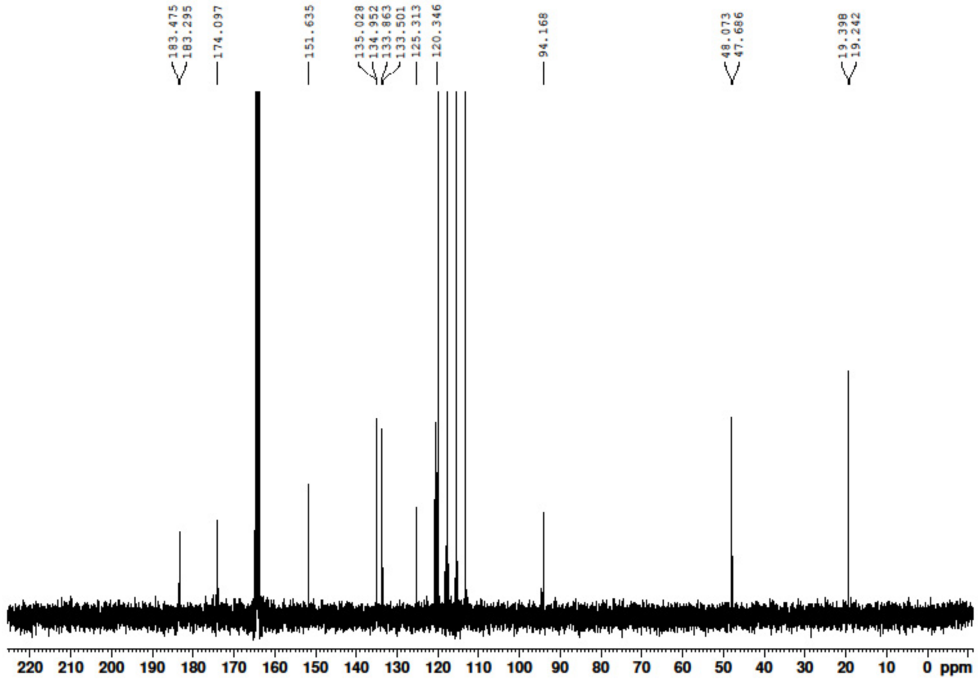

[1]**H NMR** spectrum of mixture of **$2f_A$** and **$2f_B$** in TFA-d.

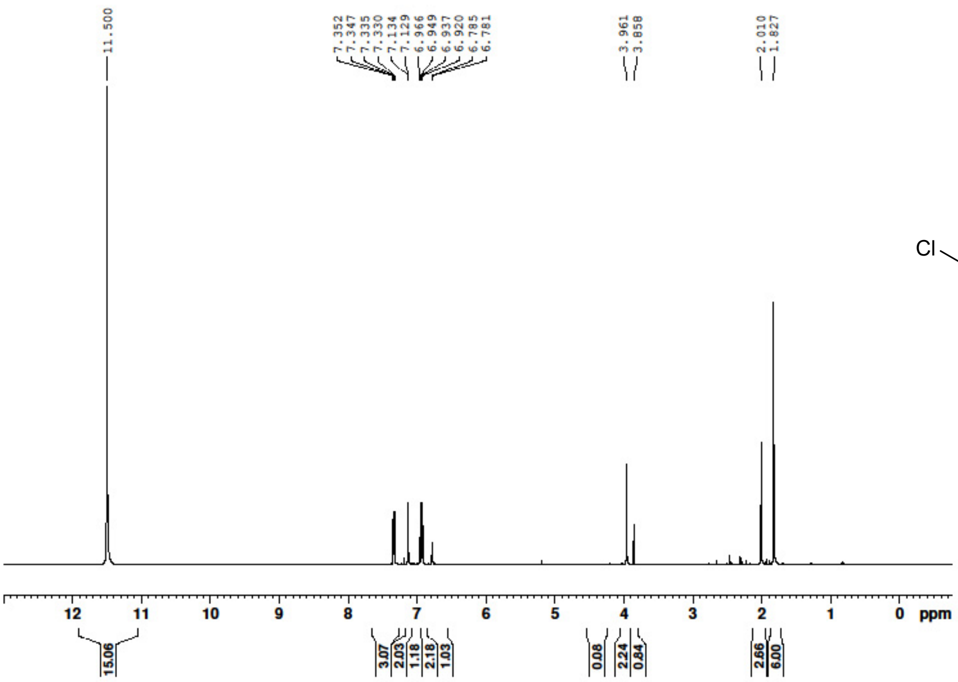

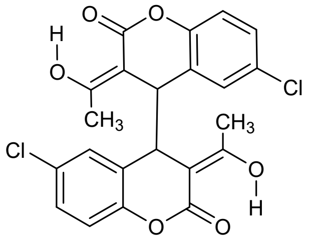

[13]**C NMR** spectrum of mixture of **$2f_A$** and **$2f_B$** in TFA-d.

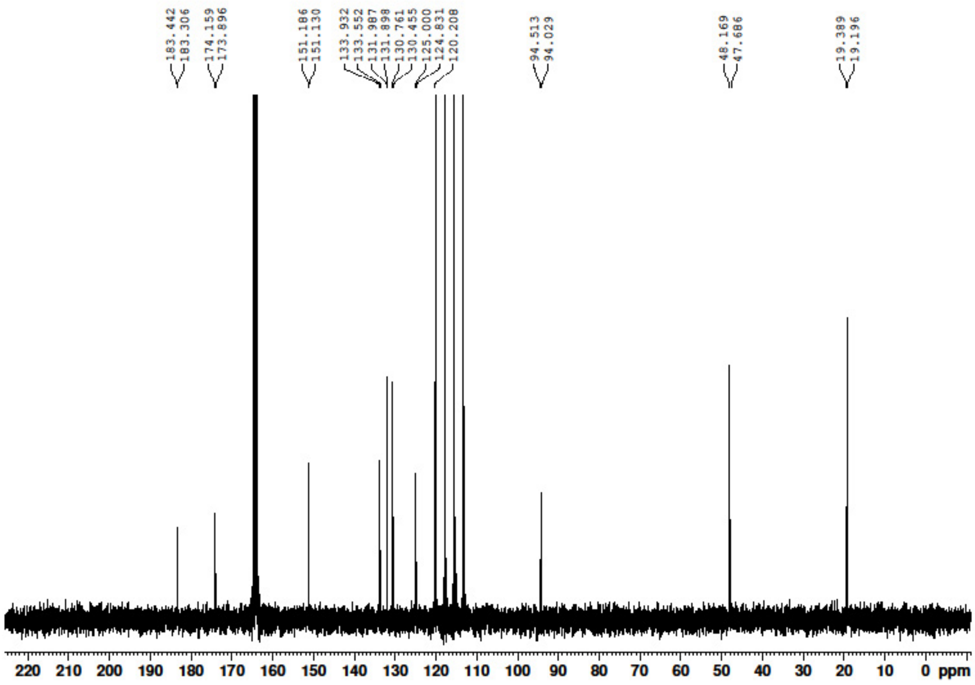

^{1}H NMR spectrum of mixture of **2g** in TFA-d.

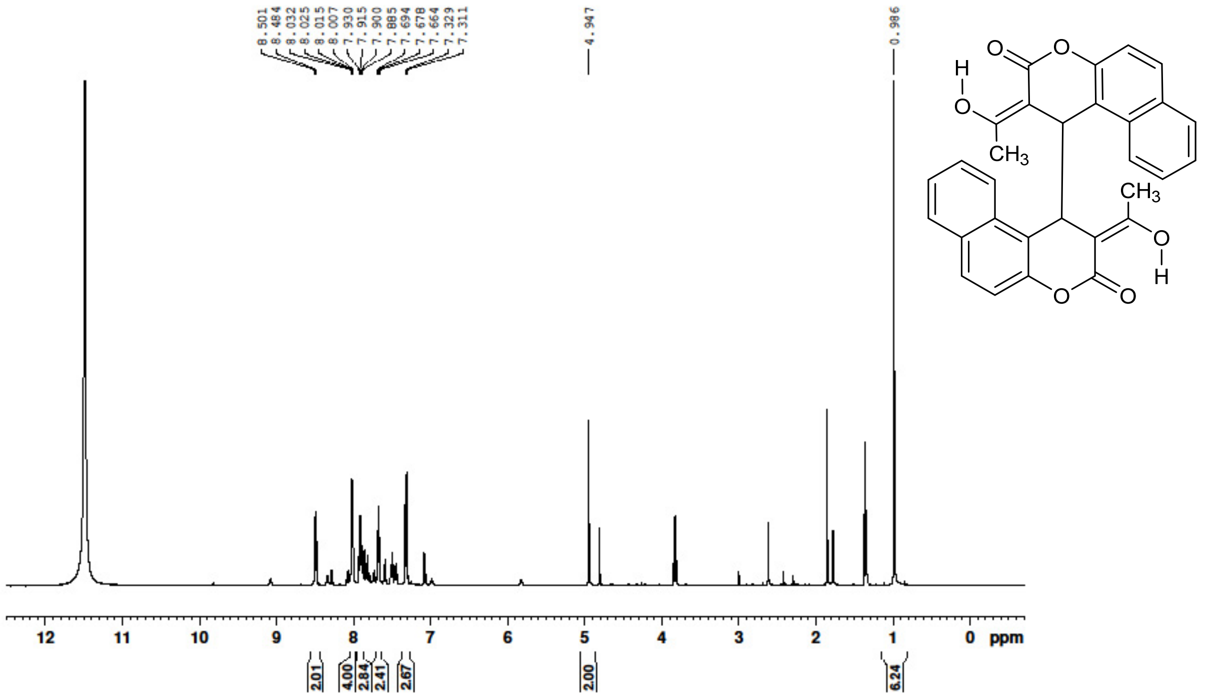

^{13}C NMR spectrum of mixture of **2g** in TFA-d.

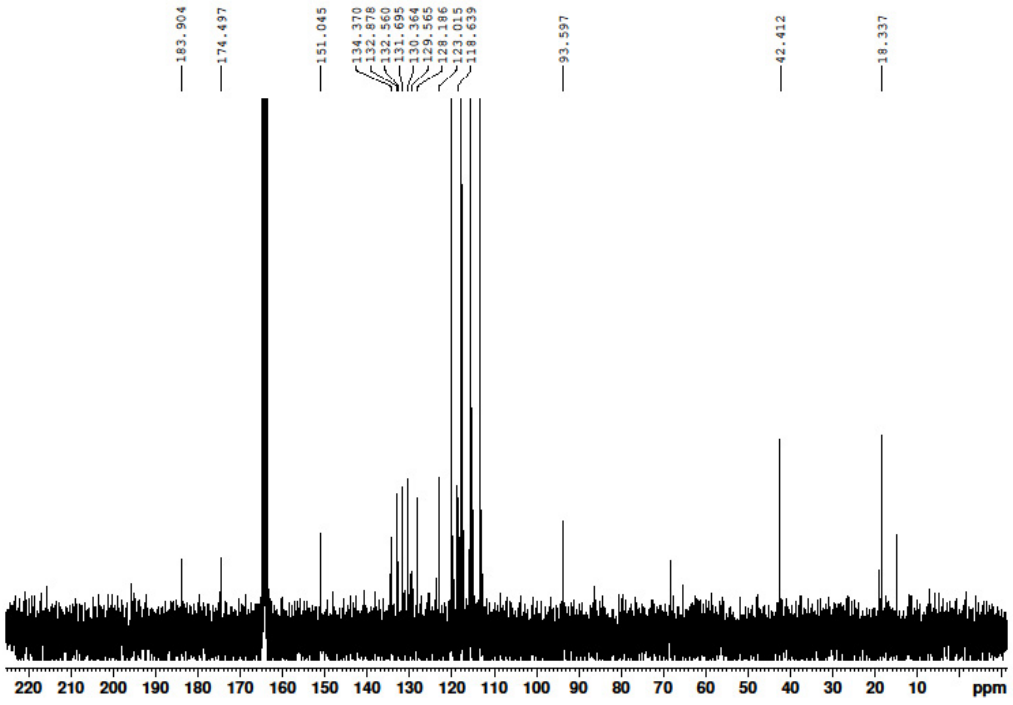

[1]**H NMR** spectrum of mixture of **2h**$_{\mathbf{A}}$ and **2h**$_{\mathbf{B}}$ in TFA-d.

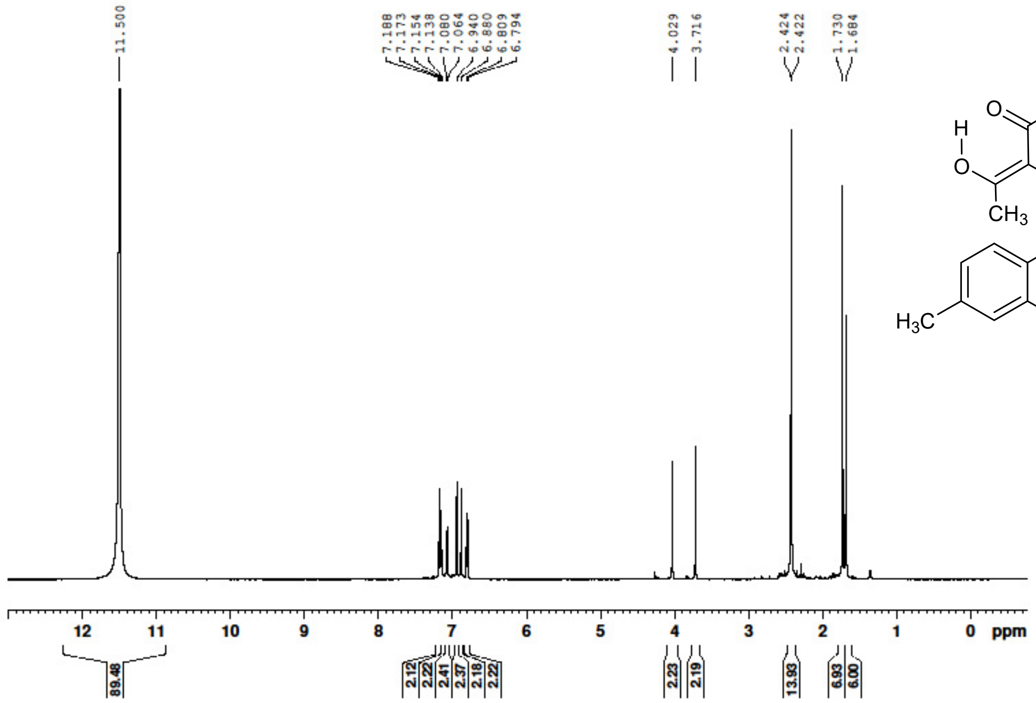

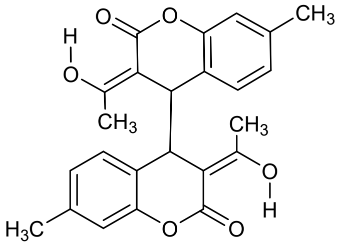

[13]**C NMR** spectrum of mixture of **2h**$_{\mathbf{A}}$ and **2h**$_{\mathbf{B}}$ in TFA-d.

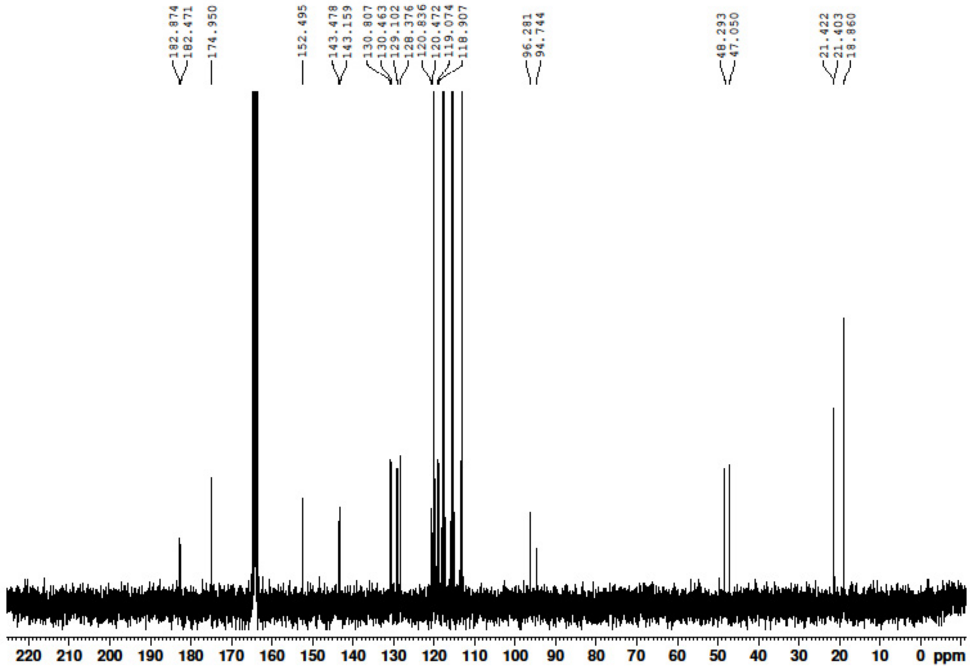

NOESY spectrum of **2h**$_A$ and **2h**$_B$ in TFA-d.

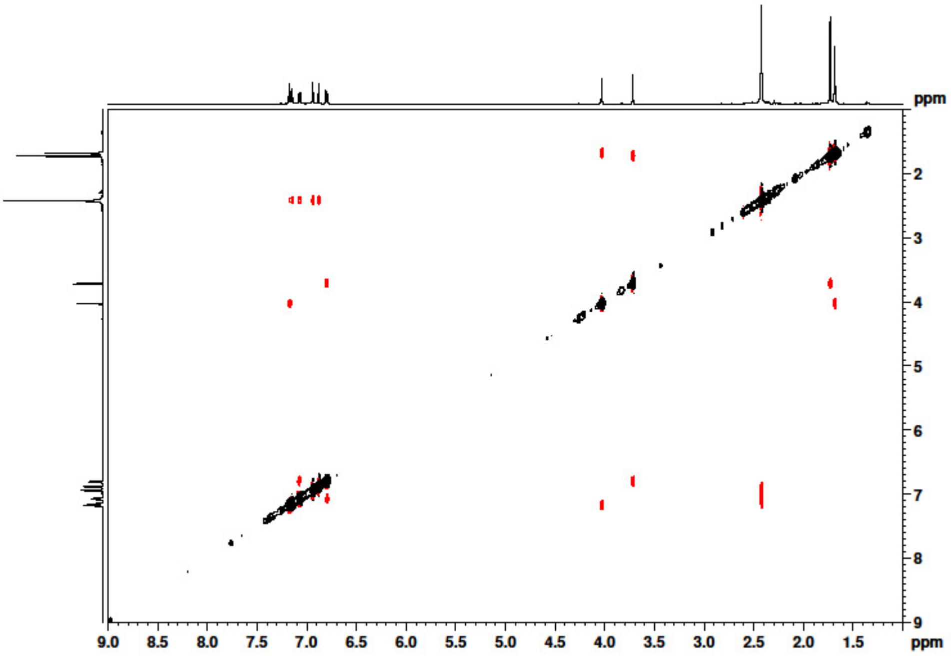

Supplement: Supplementary file 1 [file molecules-30-00651-s001.zip › molecules-3403258-supplementary 1.pdf]
